# Case Report: Contrasting BCL2 Upregulation With Venetoclax in a Case of Refractory Lymphomatoid Papulosis and Progressive Chronic Lymphocytic Leukemia

**DOI:** 10.3389/fonc.2021.729106

**Published:** 2021-09-09

**Authors:** Valerio Guarente, Giovanni Martino, Erica Dorillo, Filomena De Falco, Chiara Rompietti, Daniele Sorcini, Mariangela Brogna, Valeria Cardinali, Stefano Ascani, Andrea Marra, Paolo Sportoletti

**Affiliations:** Institute of Hematology-Centro di Ricerca Emato-Oncologica (CREO), Department of Medicine and Surgery, University of Perugia, Perugia, Italy

**Keywords:** T-cell lymphoma, chronic lymphocytic leukemia, venetoclax (BCL2 inhibitor), lymphomatoid papulosis (LyP), lymphomatoid papulosis treatment

## Abstract

A 57-year-old man affected by high-risk progressive chronic lymphocytic leukemia (CLL), primary resistant to first-line chemoimmunotherapy, developed a type A lymphomatoid papulosis (LyP) during a second progression of CLL. The two blood tumor entities were clonally unrelated. LyP presented with a diffuse (>90% body surface area) cutaneous rash and was characterized by intensely pruriginous dusky nodules (n = 10) and red flat-topped papules (n = 60). No response to topical corticosteroids and psoralen plus ultraviolet A (PUVA) phototherapy was observed. In order to effectively treat progressive *TP53*-mutated CLL, the potent BCL2 inhibitor, venetoclax, was initiated with no treatment-related complications. While CLL only achieved a partial response, a complete remission of LyP-associated cutaneous rash and of the intractable pruritus was obtained within 2 months from venetoclax initiation. BCL2 immunostaining of the original cutaneous specimen showed a strong over-expression of the anti-apoptotic protein, restricted to CD30^+^ lymphoid cells and reactive microenvironment. At 12 months follow-up, the patient is still in complete remission of LyP. Our findings underline the probable pathogenic role of BCL2 in LyP and the potential therapeutic efficacy of venetoclax for the treatment of this primary cutaneous CD30^+^ lymphoproliferative disorder, especially in the setting of severe and refractory disease.

## Introduction

Lymphomatoid papulosis (LyP) is a primary cutaneous CD30^+^ T-cell lymphoproliferative disorder characterized by chronic, recurrent, and self-healing papulonecrotic or nodular skin lesions, generally localized to the trunk and extremities (1). LyP accounts for ∼12% of cutaneous T-cell lymphomas (CTCLs) and usually exhibits a benign course (as some cases can regress spontaneously), with a 5-year disease-specific survival of 99% (1). In ∼20% of patients, LyP may be preceded by, associates with, or followed by another malignant lymphoma such as mycosis fungoides, anaplastic large T-cell lymphoma, or, more rarely, Hodgkin’s lymphoma (1); however, LyP has been anecdotally reported in patients with chronic lymphocytic leukemia/small lymphocytic lymphoma (CLL/SLL), only accounting for 11 adult cases previously described (2).

LyP has no approved therapy and can often be very difficult to treat, especially in the presence of extensive and ulcerative lesions or chronic intractable pruritus (1, 3); recently, brentuximab vedotin, a monoclonal antibody directed against CD30, has proved effective in treating refractory LyP in a phase II trial (4, 5), achieving an overall response rate in 12/12 cases [100%] and a complete response rate in 5/12 cases [58%]; however, its use in the clinic might be limited (at least in part) by peripheral neuropathy [10/12 cases (83%) and 5/12 cases (42%) experienced grade 2 neuropathy (5)].

## Case Presentation

**Figure 1:**
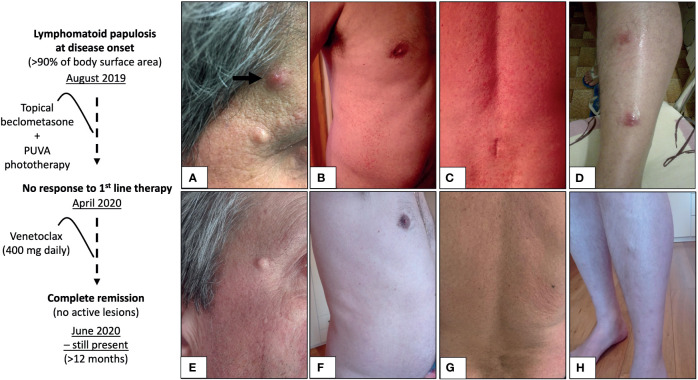
Lymphomatoid papulosis at disease onset **(A–D)** and at 8 weeks from the initiation of venetoclax monotherapy **(E–H)**. Intensely pruritic red flat-topped papules with diffuse erythema spreading on >90% body surface area **(B, C)**, accompanied with sparse nodules (**A**, black arrow, and **D**). At 8 weeks from the initiation of the potent selective BCL2 inhibitor venetoclax (400 mg daily), a complete response was achieved, as defined by no detectable active skin lesions **(E–H)**, and it is still present (in May 2021), lasting more than 12 months.

In June 2014, a 57-year-old Caucasian man was diagnosed with chronic lymphocytic leukemia (CLL), Rai stage II, Binet stage A, genetically driven by an unmutated immunoglobulin variable heavy-chain (*IGVH*) gene status, with no mutations in *NOTCH1*, *SF3B1*, and *TP53* genes; interphase fluorescence *in situ* hybridization (I-FISH) performed with the peripheral blood lymphocytes identified a IGVH/14q32 deletion and no other cytogenetic aberrations. CLL B cells did not express zeta chain-associated protein kinase 70 (ZAP70) and CD38. Blood counts showed lymphocytosis (lymphocyte absolute count: 8,500 × 10^9^/L) and no anemia or thrombocytopenia. The patient underwent a careful clinical and laboratory follow-up for ∼4.5 years. In January 2019, CLL progressed with notable lymphocytosis (331,000 × 10^9^/L) and macrocytic anemia [Hb 11.2 g/dl, mean corpuscular volume (MCV) 112 fl], accompanied with supra-/subdiaphragmatic lymphadenopathies (>3 regions) and splenomegaly (longitudinal spleen diameter of 22 cm); no *TP53* mutations/deletion underlaid disease progression. Chemoimmunotherapy with intravenous bendamustine (90 mg/m^2^, days 1 and 2) and rituximab (375 mg/m^2^, day 1) was then administered (for 6 cycles, every 28 days, from January to July 2019), achieving a partial response (6). In August 2019, the patient presented with a skin rash of intensely pruritic dusky nodules (n > 10) and red flat-topped papules (n > 300) with erythema involving >90% of body surface area (**Figures 1A–D**).

**Figure 2:**
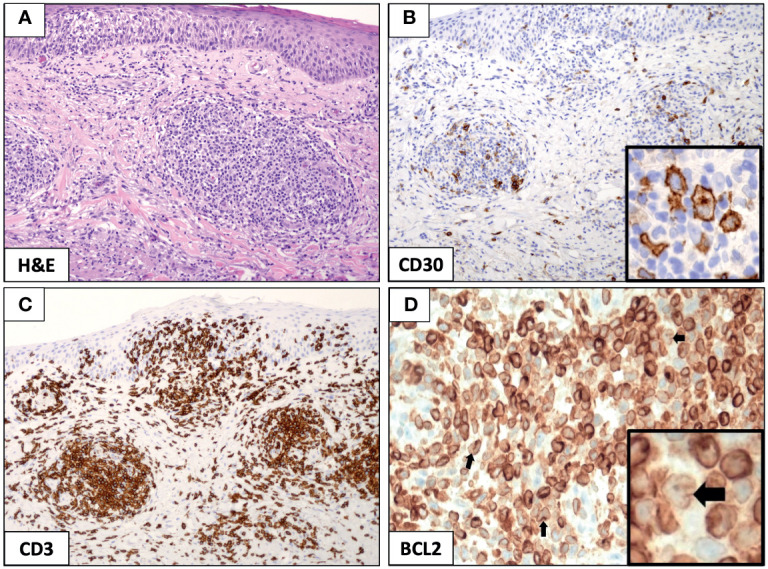
Immunohistochemical analysis of mixed-infiltrate type A lymphomatoid papulosis (LyP). The punch biopsy from the left arm identified a diffuse infiltration of small lymphocytes and few large-sized Hodgkin-like cells that were prevalent in the dermis and subcutis, while largely sparing the epidermis (**A**, *original magnification*, ×10). Hodgkin-like cells stained positive for CD30 (**B**, *original magnification*, ×10; and B, inset, *original magnification*, ×40) and were interspersed in a dense lymphoid infiltrate mostly contributed by CD3^+^ T cells (**C**, *original magnification*, ×10). The expression of the anti-apoptotic protein BCL2 was strong and pervasive in the LyP tissue (**D**, *original magnification*, ×10), and it was readily recognized in the reactive lymphoid meshwork and large-sized Hodgkin-like cells (**D**, inset, black arrow, *original magnification*, ×40).

The punch biopsy specimen obtained from the left arm and stained with hematoxylin and eosin demonstrated an abnormal mixed-lymphoid infiltrate (composed of small lymphocytes and few large-sized Hodgkin-like cells) in the dermis and subcutis, with moderate extension into epidermis (**Figure 2A**). Immunohistochemical analysis showed that pathological Hodgkin-like cells were CD3^+^, CD4^+^, CD30^+^ (**Figure 2B**) and CD8^−^, and were intermingled with numerous inflammatory cells (mostly of T-cell origin; **Figure 2C**); no monomorphic small neoplastic B cells nor immunoglobulin gene rearrangements were detected in the skin biopsy, thus excluding a diagnosis of B-cell leukemia cutis while proving that the CD30^+^ cutaneous lymphoproliferative disorder was clonally unrelated to the mature B-cell neoplasm in our case. In conclusion, the clinicopathological picture was consistent with mixed-infiltrate type A LyP (1).

Topical corticosteroid beclomethasone (from August 2019 to January 2020) and psoralen plus ultraviolet A (PUVA) phototherapy were administered (three times per week, from January to April 2020) with no improvement of the diffuse skin papulonodular rash nor of the intractable pruritus associated with scratch lesions [pruritus was defined as of severe entity, according to a visual analogue scale (7)]. In April 2020, a second progression of CLL occurred and it was clinically characterized by progressive lymphadenopathy and, genetically, by the acquisition of a pathogenic nonsense mutation (*TP53* ^E294X^) and of a monoallelic deletion of *TP53* gene (del*TP53*/17p11). A salvage therapy with oral venetoclax (400 mg, daily, following the 5-week ramp-up dosing schedule), a potent selective BCL2 inhibitor, was then started and induced a partial response of CLL (6) with no treatment-related complications (in particular, no tumor lysis syndrome or neutropenia); owing to the risk of cardiotoxicity, the administration of Bruton’s tyrosine kinase inhibitor, ibrutinib, was not considered in this patient who had recently suffered acute myocardial infarction. Very surprisingly, LyP-associated skin lesions completely resolved (zero lesions) along with intractable pruritus within 8 weeks from venetoclax initiation (**Figures 1E–H**).

## Discussion

In order to search for the molecular underpinning of patient’s dramatic response to venetoclax, we stained the original cutaneous biopsy for the BCL2 anti-apoptotic protein and demonstrated its strong expression restricted to the CD30^+^ Hodgkin-like cells and reactive lymphoid infiltrate (**Figure 2D**); a positive staining for BCL2 has been previously described in 100% of LyP cases studied by immunohistochemistry [8 and 5 cases from two independent studies (8, 9)], and it has been mostly observed within the reactive small T-cell infiltrate of the LyP tissue (13/13); however, only in 1/8 cases (∼12%), BCL2 over-expression has been even detected in the Hodgkin-like cell component (9), as it is for the current case. Notably, BCL2 protein and mRNA expression have shown to have an inverse relationship with *in vitro* sensitivity to venetoclax in primary cells from patients with CTCL, and a higher BCL2 expression at baseline does correlate with a better response to venetoclax (10); these observations are in line with the patient’s durable complete response to venetoclax (lasting >12 months from drug initiation and still present in May 2021), which is defined as no detectable active skin lesion (11).

## Concluding Remarks

Our findings underline the probable pathogenic role of BCL2 in LyP and the potential therapeutic efficacy of the BCL2 inhibitor, venetoclax, for the treatment of this primary cutaneous CD30^+^ lymphoproliferative disorder, especially in the setting of severe and refractory disease.

## Data Availability Statement

The original contributions presented in the study are included in the article, further inquiries can be directed to the corresponding authors.

## Ethics Statement

Ethical review and approval were not required for the study on human participants in accordance with the local legislation and institutional requirements. The patients/participants provided their written informed consent to participate in this study. Written informed consent was obtained from the individual(s) for the publication of any potentially identifiable images or data included in this article.

## Author Contributions

VG, AM, and PS conceived the study. VG, VC, MB, and PS were involved in patient’s clinical management and treatment. VG, GM, SA, AM, and PS performed the pathological examination of the skin biopsy. ED, FDF, CR, and DS performed molecular genetic analyses. VG, AM, and PS wrote the paper. AM and PS supervised the entire study. All authors contributed to the article and approved the submitted version.

## Funding

This research has received funding from the Associazione Italiana per la Ricerca sul Cancro (AIRC) IG 2018, Project ID 21352, to PS.

## Conflict of Interest

The authors declare that the research was conducted in the absence of any commercial or financial relationships that could be construed as a potential conflict of interest.

## Publisher’s Note

All claims expressed in this article are solely those of the authors and do not necessarily represent those of their affiliated organizations, or those of the publisher, the editors and the reviewers. Any product that may be evaluated in this article, or claim that may be made by its manufacturer, is not guaranteed or endorsed by the publisher.
